# Overexpression of candidate tumor suppressor ECRG4 inhibits glioma proliferation and invasion

**DOI:** 10.1186/1756-9966-29-89

**Published:** 2010-07-04

**Authors:** Wei Li, Xinrui Liu, Bo Zhang, Dongxue Qi, Lihong Zhang, Yuhong Jin, Hongfa Yang

**Affiliations:** 1 The Key Laboratory of Pathobiology, Ministry of Education, Jilin University, 130021, PR China; 2 Department of Neurosurgery, the First Affiliated Hospital of Jilin University, 130021, PR China

## Abstract

**Background:**

ECRG4 has been shown to be a candidate tumor suppressor in several tumors, but its role in glioma remains poorly understood. In this study, we examined the mRNA expression of ECRG4 and investigated its biological role in glioma cells.

**Methods:**

Real-time PCR was used to examine expression of ECRG4 in gliomas and their matched brain tissues. The effect of ECRG4 expression on cell proliferation, invasion, and migration was investigated in human U251 glioma cells. Finally, the regulation of transcription factor NF-kB by ECRG4 was evaluated by western blotting.

**Results:**

Of the 10 paired samples analyzed, 9 glioma tissues displayed the decreased expression of ECRG4 compared to matched normal brain tissues. Cells transfected with ECRG4 showed significantly decreased cell proliferation as evaluated by MTT and colony formation assays. Furthermore, overexpression inhibited cell migration and invasion in transwell and Boyden chamber experiments and retarded the cell cycle progression from G1 to S phase by FACSCaliber cytometry. Protein levels of nuclear transcription factor NF-kB, which is involved in cell proliferation, inversely correlated with ECRG4 expression.

**Conclusion:**

Our data suggest that ECRG4 serves as a tumor suppressor in glioma.

## Introduction

Glioma is the most common and aggressive form of brain tumors that affects adults. Despite advances in surgical and clinical neuro-oncology, malignant glioma prognosis remains poor due to its diffuse and invasive nature. To date, the molecular pathogenesis of glioma is still unclear. As a result, a major research effort has been directed at identification of specific genes which might play important roles in glioma carcinogenesis.

The ECRG4 gene [GenBank accession no.AF325503] was initially identified and cloned by Bi *et al*[1,2] by comparing differential gene expression between human normal esophageal epithelia and ESCCs from high incidence families in Linxian County of Northern China. Further, this group [3,4] and Mori [5] found that ECRG4 expression was significantly decreased in ESCC tissues and cell lines compared to normal adult esophageal epithelia.

Hypermethylation of CpG islands of gene promoter often causes transcriptional silencing of genes, including tumor suppressor genes [6-10]. Previous studies reported promoter hypermethylation and reduced expression of *ECRG4 *in advanced esophageal, prostate carcinomas, colorectal carcinoma, and glioma[3,11,12] Together with a study in esophageal cancer cell lines[4], these reports suggest that ECRG4 may play a tumor suppressor role in certain cancers including glioma. However, the function and mechanisms mediated by the loss of ECRG4 expression in glioma remains unclear.

In the present study, we examined the expression of ECRG4 in gliomas and explored its role as a tumor-suppressor gene in glioma cells *in vitro*. We provided a preliminary molecular mechanism of ECRG4-mediated suppression of glioma cell growth.

## Materials and methods

### Cells

U251 human glioma cells were cultured in RPMI1640 medium (HyClone Inc, USA) supplemented with 10% new calf bovine serum (NCBS) (PAA Laboratories, Inc, Austria) in a 37°C, 5% CO_2 _incubator.

### Sample collection

Ten (10) fresh paired gliomas and adjacent normal brain were collected from the first Affiliated Hospital of Jilin University, China, at the time of first resections before any therapy. All fresh samples were immediately preserved in liquid nitrogen. Prior consent from patients and approval from the Ethics Committees of this hospital were obtained for use of these clinical materials for research purposes. All specimens had confirmed pathological diagnosis.

### Real-time PCR

Real-time PCR was performed to measure the expression of ECRG4 mRNA using SYBR Premix Ex Taq (Takara, Japan) with an Mx3000P real-time PCR system (Stratagene, La Jolla, CA, USA) as described previously [13]. The sequence for sense primer was 5'- TTCCTTGGCAGCCTGAAGCG-3', and for antisense primer was 5'- GGCTCCATGCCTAAAGCCGT-3'. *GAPDH *gene was used as an internal control using the sense primer 5'-GCACCGTCAAGGCTGAGAAC-3' and antisense primer 5'-TGGTGAAGACGCCAGTGGA-3'.

### Construction of pECRG4-EGFP-N1 vector and Establishment of glioma U251 cell line stably expressing ECRG4

The ECRG4 open reading frame was amplified from cDNA clone IMAGE:5260075 using the forward primer 5'- *ATAC***GTCGAC**ATGGCTGCCTCCCCCGCG-3' and the reverse primer 5'-*CGAT***GGATCC**GTAGTCATCGTAGTTGACGCT-3' introducing SalI and BamHI restriction endonuclease sites. ECRG4 cDNA digested with SalI and BamHI was cloned into a pEGFP-N1 eukaryotic expression vector. The resulting vector was transfected into U251 cells using lipofectamine 2000 (Invitrogen, Carlsbad, CA). An "empty" vector pEGFP-N1 was utilized as a negative control. After 24 to 48 h, the transient transfection efficiency was determined using an Olympus fluorescence microscope. Cells were then passaged at appropriate ratios in six-well plates. The next day, cells were cultured in the presence of 1,000 to 2,000 μg/mL G418 (Life Technology) increased in a stepwise manner for 14 days for selection of highly expressing GFP cells. Total RNA of all single cell clones was isolated and quantitative RT-PCR performed to detect the mRNA level of ECRG4 as described above. Each sample was measured at least three times.

### Western blot analysis

Approximately 5 × 10^6 ^U251 cells were lysed in RIPA Buffer and total protein concentration determined with BCA assay (Beyotime Inc, China). Total protein (30 μg) was loaded onto 12% SDS-PAGE gel. Antibodies used for Western blot analysis included: polyclonal anti-GFP antibody (Abcam, MA, USA, 1:400), NF-kB (Abcam, MA, USA, 1:400), and anti-ACTB antibody (Santa Cruz, USA, 1:400), and HRP-conjugated anti-rabbit secondary antibody (Zhongshan Inc, 1:2000). Each experiment was performed in triplicate.

### Cell proliferation analysis

Cell growth was determined by MTT assay (Sigma, USA). Briefly, 1 × 10^3 ^cells were seeded into 96-well plate in quadruplicate for each condition. Approximately 72 h later, MTT reagent was added to each well at 5 mg/mL in 20 μL and incubated for another 4 h. The formazan crystals formed by viable cells were then solubilized in DMSO and measured at 490 nm for the absorbance (A) values. Each experiment was performed in triplicate.

### Plate colony formation assay

Approximately 100 cells were added to each well of a six-well culture plate. After incubation at 37°C for 15 days, cells were washed twice with PBS and stained with Giemsa solution. The number of colonies containing ≥50 cells was counted under a microscope [plate clone formation efficiency = (number of colonies/number of cells inoculated) × 100%]. Each experiment was performed in triplicate.

### Cell Cycle analysis

Cells grown in regular growth or serum-free media for 36 h were collected, fixed in methanol and stained with PBS containing 10 μg/mL propidium iodide and 0.5mg/mL RNase A for 15 min at 37°C. The DNA content of labeled cells was acquired using FACS Caliber cytometry (BD Biosciences). Each experiment was performed in triplicate.

### In Vitro migration and Invasion assay

Cells growing in the log phase were treated with trypsin and re-suspended as single-cell solutions. A total of 1 × 10^5 ^cells were seeded on a fibronectin-coated polycarbonate membrane insert in a transwell apparatus (Corning Inc, USA). In the lower chamber, 600 μl RPMI 1652 with 10% NBCS added as a chemoattractant. After the cells were incubated for 14 h at 37°C and 5% CO_2 _incubator, the insert was washed with PBS, and cells on the top surface of the insert were removed by a cotton swab. The matrigel invasion assay was similar to the cell migration assay, except the transwell membrane was precoated with ECMatrix and the cells were incubated for 16 hours at 37°C and 5% CO_2 _incubator. Cells adhering to the lower surface were fixed by methanol, stained by Giemsa and counted under a microscope in five predetermined fields (×200). All assays were independently repeated at least three times.

## Results

### Downregulated expression of ECRG4 in Gliomas

**Figure 1:**
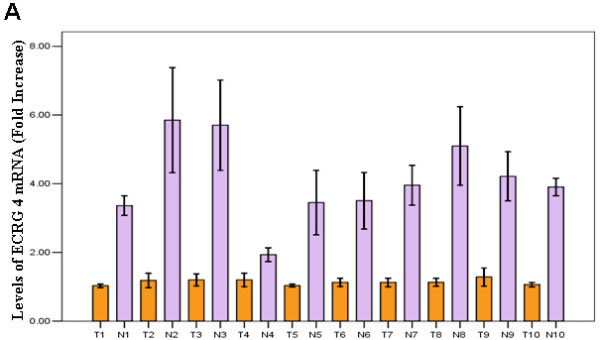
**The reduced expression levels of *ECRG4 *mRNA in glioma. A. **ECRG4 mRNA level was markedly downregualted in glioma tissue comparing to their matched normal brain tissues. (T: Tumor; N: Normal tissue).

In order to assess the role of ECRG4 in glioma, we performed real-time PCR to measure the expression of ECRG4 mRNA transcripts in 10 paired gliomas and their adjacent brain tissues. As shown in Figure 1A, 9 glioma tissues showed markedly decreased expression (>2-fold change) of ECRG4 compared to their matched normal tissues.

### Overexpression of ECRG4 in glioma U251 cell line

**Figure 2:**
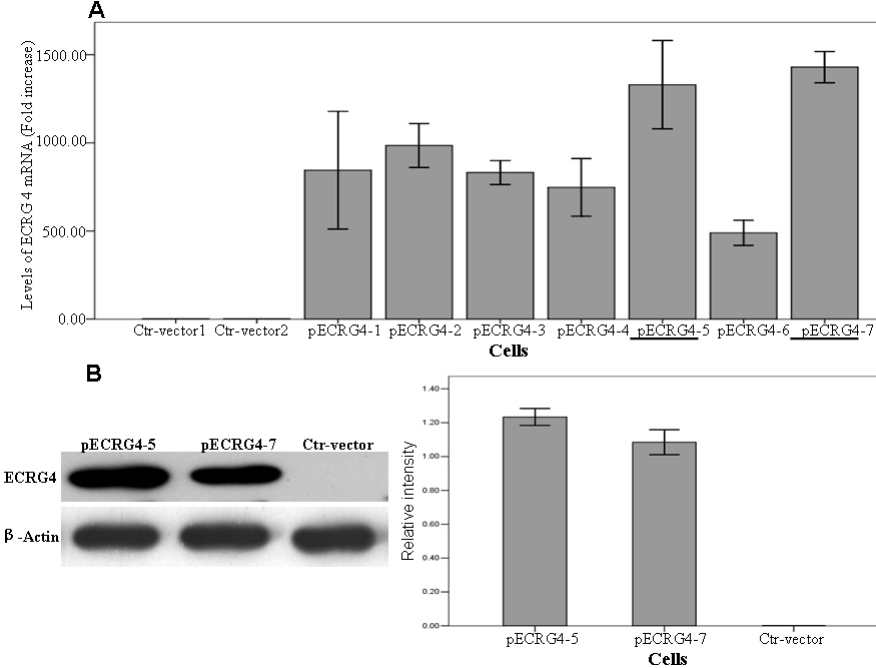
**Restored expression of *ECRG4 *in glioma U251 cells. A. **Real-time PCR analysis indicated the highest mRNA expression of *ECRG4 *in two cell clones pEGFP-ECRG4-5 and -7. **B. **Western blotting assay shows significantly increased protein expression of *ECRG4 *in pEGFP-ECRG4-5 and -7 comparing to Control cells. β-actin was used as the internal control.

To study the biological functions of *ECRG4*, we introduced *ECRG4 *into U251 glioma cells using a pEGFP-N1 eucaryotic expression vector containing *ECRG4 *gene. Seven stably transfected cell clones were obtained. Real-time PCR identified two cell clones(ECRG4-5,-7) with the highest mRNA expression of *ECRG4*(Figure 2A). Further, Western blotting assay with a GFP antibody showed that *ECRG4-GFP *fusion protein in two cell clones was highly expressed, compared to control clone cells (Figure 2B).

### ECRG4 inhibits cell proliferation *in vitro*

**Figure 3:**
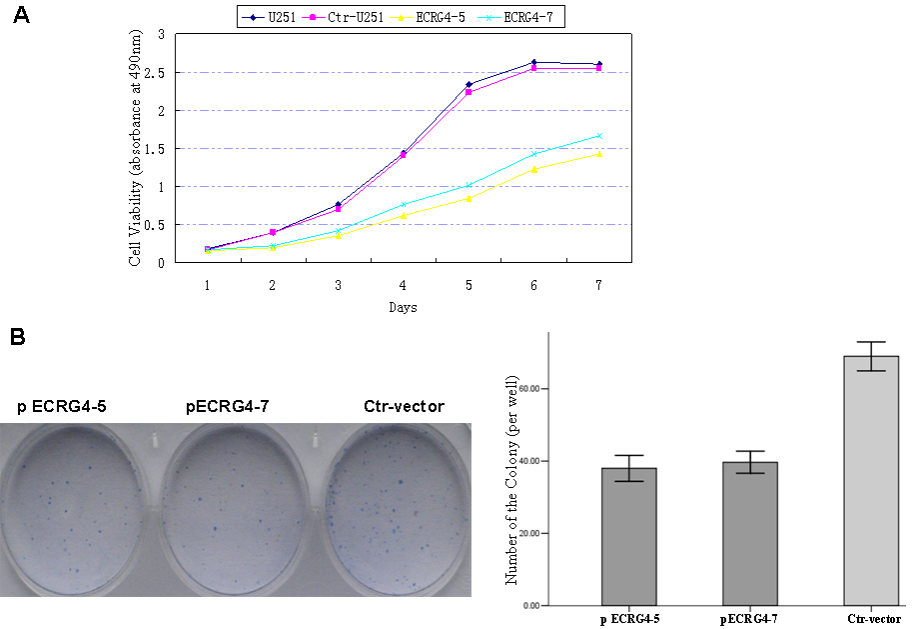
**Overexpression of *ECRG4 *inhibted cell proliferation *in vitro***. **A**. The cell growth of parental U251 cells, Control-vector cells and pEGFP-ECRG4-5 and -7 cells, were examined by MTT assay over a seven-day period. *P < 0.05, as compared to U251 and Control-vector cells. **B. **The cell growth of Control-vector cells and pEGFP-ECRG4-5 and -7 cells, were examined by plate colony formation assay. *P < 0.05, as compared to U251 and Control-vector cells.

To analyze the function of *ECRG4*, we studied the rate of cell proliferation of *ECRG4*-expressing ECRG4-5 and -7 cells. The growth curves determined by an MTT assay showed that ECRG4 significantly inhibited cell proliferation of these two lines of cells compared to parental line U251 and Control clone cells (Figure 3A). The results from a colony formation assay showed that *ECRG4*-overexpressing ECRG4-5 and -7 cells formed significantly less colonies than *Control *clone cells (*P *< 0.001 for both cell types) (Figure 3B), suggesting an inhibitory effect of ECRG4 on anchorage-dependent growth of glioma cells.

### ECRG4 suppressed cell migration and invasion

**Figure 4:**
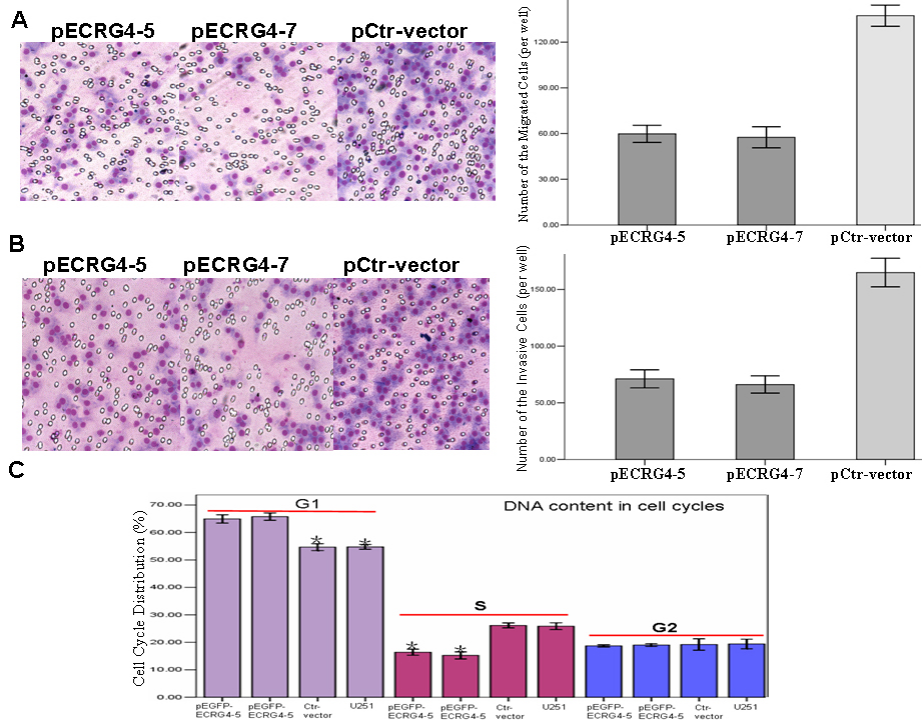
**Increased *ECRG4 *expression inhibited cell migration, invasion and cell cycle progression**. (**A**) Cell migration and (**B**)invasion capabilities of Control-vector cells, pEGFP-ECRG4-5 and -7 cells, were examined using transwell assay and boyden chamber assay. Data were presented as mean ± SD for three independent experiments. *P < 0.05, as compared to Control-vector cells. C. Cell cycle in parental U251 cells, Control-vector cells and pEGFP-ECRG4-5 and -7 cells, was determined by FACS Caliber cytometry. *P < 0.05, as compared to parental U251 cells and Control-vector cells

To measure the effect of ECRG4 on cell migration, ECRG4-expressing ECRG4-5 and -7 cells were cultured on a transwell apparatus. After 12-h incubation, cell migration was significantly decreased in both ECRG4-overexpressed cell groups compared to the parental U251 cells and the ECRG4-negative control cells (for both P < 0.001) (Figure 4A). Using a Boyden chamber coated with matrigel, we measured cell invasion after 16-h incubation. Compared with the negative control cells, ECRG4-expressing -5 and -7 cells both showed significantly decreased invasiveness (for both *P *< 0.001) (Fig 4.B).

### Inhibition of cell cycle by ECRG4

To detect the effect of *ECRG4 *on the cell cycle, we measured cell cycle distribution in *ECRG4*-expressing -5 and -7 cells. In these lines the S-phase population was markedly decreased while the G1 population significantly increased in both two cell lines compared to the Ctr-vector cells and U251 cells (*P *< 0.001). Neither cell line had significant changes in the G2 population (Figure 4C)(Table 1).

**Table 1:** Overexpressed ECRG4 retarded cell cycle progression from G1 to S phase

Cells	G1	S	G2
pEGFP-ECRG4-5	64.93 ± 1.54	16.37 ± 1.12	18.7 ± 0.44
pEGFP-ECRG4-7	5.77 ± 1.34	15.23 ± 1.30	19.0 ± 0.44
Ctr-Vector	54.67 ± 1.27	26.13 ± 0.91	19.2 ± 2.05
U251	54.73 ± 0.86	25.87 ± 1.27	19.4 ± 1.77

### ECRG4 inhibited the expression of NF-Kb

**Figure 5:**
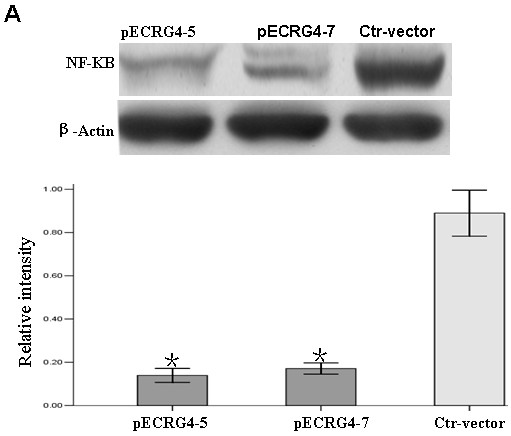
**Overexpresed *ECRG4 *expression suppressed the expression of *NF-kB *protein. A. **Protein expression of *NF-kB *was decreased in pEGFP-ECRG4-5 and -7 cells compared to Control-vector cells. Data were presented as mean ± SD. *P < 0.05.

We were further interested in exploring the molecular mechanism of ECRG4 tumor-suppression in glioma. We found that restoration of ECRG4 expression in glioma U251 cells inhibited expression of transcription factor NF-κB (Figure 5A). This suggested that ECRG4 may be involved in NF-κB pathway in glioma.

## Discussion

Malignant glioma is a highly invasive and clinically challenging tumor of the central nervous system, and its molecular basis remains poorly understood. We became interested in ECRG4 because it is normally expressed in the brain yet was found to be downregulated in gliomas. Northern blot assays revealed that ECRG4 is also expressed in other tissues including heart, placenta, lung, liver, skeletal muscle, kidney and pancreas [14]. Further, ECRG4 promoter hypermethylation has been attributed to decreased expression in esophageal, prostatic, and colorectal cancers. Together these results suggest that ECRG4 might play a suppressive role in tumor pathogenesis. ECRG4 contains a 772-bp full-length cDNA fragment, and its open reading frame is 444bp encoding a 148-amino acid polypeptide with molecular weight of 17 kDa. ECRG4 gene is located at chromosome 2q12.2 and contains 4 exons spanning about 12,500 bp.

In order to assess the role of ECRG4 in glioma, we first performed real-time PCR to measure the expression of ECRG4 mRNA transcripts in 10 paired gliomas and their adjacent brain tissues. Similar to observations by Götze *et al *[12], we found that ECRG4 expression was significantly downregulated in 9 glioma tissues compared to their matched normal tissues.

To examine whether ECRG4 plays a suppressive role in glioma pathogenesis, we applied a gain-of-function approach by introducing ECRG4 into cells to investigate its biological function. To this end, we chose the U251 glioma cell line which exhibits relatively low expression level of endogenous ECRG4 (data not shown) and provides a biologically relevant model for our study. U251 cells were transfected with ECRG4-GFP-expressing eukaryotic vector followed by selection with G418. We successfully established lines stably expressing ECRG4 protein at dramatically elevated levels compared to control cells. Subsequent functional studies demonstrated that overexpression of ECRG4 led to significantly reduced *in vitro *cell growth and G1/S transition blockage. This is consistent with findings by Li *et al *[4,12] that showed up-regulation of ECRG4 inhibited cell proliferation and cell cycle progression. This suggests that the biological functions of ECRG4 are not unique to a specific cancer type, but likely common among multiple cancers. Our study has revealed a novel function of ECRG4 in suppression of glioma cell migration and invasion, implicating its potential involvement in cancer metastasis. This hypothesis should to be further validated in an *in vivo *animal model. The observation that ECRG4 regulates multiple cellular processes such as cell growth, cell cycle, migration, and invasion in multiple cancers implies it is an important therapeutic target for multiple human cancers, including glioma.

NF-kB is a transcription factor that plays a key role in carcinogenesis by controlling expression of several oncogenes, tumor suppressor genes, growth factors and cell adhesion molecules [15-17]. Li *et al *[4] previously reported that ECRG4 overexpression could suppress endogenous expression of the nuclear factor (NF-kB), which may have contributed to inhibition of esophageal cancer cell growth. Based on their finding, we speculated ECRG4 might also be involved in glioma cell growth suppression by regulating the NF- B pathway. Consistent with this hypothesis, we showed that overexpression of ECRG4 in glioma U251 cells markedly downregulated expression of NF-κB by western blot. However, further investigation is necessary to determine the exact role of ECRG4 in the NF-κB pathway within the context of glioma.

In conclusion, we found that the ECRG4's role as a tumor suppressor was supported by our observation that its expression is decreased in glioma. Furthermore, we applied gain-of-function approach to examine the biological processes regulated by ECRG4 in glioma cells. We demonstrated the functional importance of ECRG4 in suppression of glioma cell growth, migration, and invasion. Finally, we found that overexpression of ECRG4 could inhibit expression of NF-kB which may provide a mechanism explaining ECRG4's role in controlling glioma cell proliferation.

## Competing interests

The authors declare that they have no competing interests.

## Authors' contributions

WL carried out cell culture, gene transfection, gene function assays, qRT-PCR assay, and western blotting. XL, BZ, DQ, LZ, and YJ analyzed and interpreted data. HY supervised experimental work and wrote the manuscript. All authors read and approved the final manuscript.
